# High-Performance Organic Field-Effect Transistors of Liquid Crystalline Organic Semiconductor by Laser Mapping Annealing

**DOI:** 10.3390/ma17061395

**Published:** 2024-03-19

**Authors:** Luying Huang, Fenghua Liu, Jiachen Bao, Xiaoman Li, Weiping Wu

**Affiliations:** 1 Key Laboratory for Micro-Nano Physics and Technology of Hunan Province, College of Materials Science and Engineering, Hunan University, Changsha 410082, China; luyinghuang@siom.ac.cn; 2 Laboratory of Thin Film Optics, Shanghai Institute of Optics and Fine Mechanics, Chinese Academy of Sciences, Shanghai 201800, China; 21723672@shu.edu.cn (J.B.); lixiaoman@siom.ac.cn (X.L.); 3 State Key Laboratory of High Field Laser Physics, Shanghai Institute of Optics and Fine Mechanics, Chinese Academy of Sciences, Shanghai 201800, China

**Keywords:** laser annealing, liquid crystal organic semiconductors, organic field-effect transistors, mobility

## Abstract

Organic semiconductors (OSCs), especially small molecule semiconductors, have received increasing attention due to their good designability and variability. Phase transitions and interfacial properties have a decisive influence on device performance. Here, 2-Dodecyl-7-phenyl[1]benzothieno[3,2-*b*][1]benzothiophene (Ph-BTBT-12) devices are treated with low-power laser annealing, which is able to avoid the influence of the dewetting effect on the hole mobility of organic semiconductor materials. Ultraviolet ozone treatment and self-assembled monolayer treatment can improve the performance and stability of the device. Moreover, after low-temperature thermal annealing, the hole mobility of the device can even reach as high as 4.80 cm^2^ V^−1^ s^−1^, and we tested the optical response of the device to the ultraviolet wavelength and found that its maximum optical responsivity was 8.2 AW^−1^.

## 1. Introduction

Compared with traditional inorganic semiconductors, organic semiconductors (OSCs) have unique characteristics such as good flexibility, low-temperature solution processing, light weight, and adjustable optoelectronic performances, and they are also expected to be widely used in future optoelectronic devices. Based on excellent properties, low cost, and wide manufacturability, organic field-effect transistors (OFETs) are the key components for flexible electronic devices and circuits [1,2]. The organic semiconductors are often manufactured as a thin film layer between the dielectric layer and the electrodes in an OFET device [3], which is a three-terminal electronic device that includes the source, drain, and gate [4].

Organic semiconductors are categorized into small-molecule semiconductors and polymer semiconductors. The physicochemical properties of small molecules could be efficiently and simply regulated by their molecular structure, enabling them to be an ideal candidate in organic electrical and optoelectronic devices [5,6,7]. The organic semiconductors based on liquid crystalline molecules benzothieno[3,2b][1]benzothiophene (BTBT) cores have attracted much attention due to the high performance in OFETs and the solution method fabrication [8]. BTBT herein is an organic small-molecule material containing four consecutive aromatic rings, in which the S atom provides two π-electrons to form an aromatic six-electron body. Given that S atoms can stabilize the highest occupied molecular orbital (HOMO), the lower the HOMO energy level, the less likely the material is to undergo redox reactions, thus ensuring the stability of BTBT and its derivative materials in air [9,10,11]. The organic semiconductors based on liquid crystalline molecules’ BTBT cores have attracted much attention due to the high performance in OFETs and the solution method fabrication [12,13]. The organic semiconductor material 2-dodecyl-7-phenyl[1]benzothieno[3,2-*b*][1]benzothiophene (Ph-BTBT-12) has an extended conjugated structure with long alkyl chains, and proper alkylation can lead to a more compact arrangement of the conjugated skeleton, thus improving the mobility of the carriers [14,15]. Moreover, the characteristics of the active material itself and the subsequent treatments in the device preparation also play key roles in enhancing the device’s capabilities, including interface modification, annealing, and so on [16,17,18]. A self-assembled monolayer (SAM) is commonly applied in surface modification to modify the affinity energy [3,19,20,21].

Due to the high quality of energy concentration, wavelength, and pulse control, the laser has a high prospect in the fields of information transmission, micro- and nano-processing [22,23,24], and so on. Laser annealing, in particular, is a very special kind of heat treatment frequently used for semiconductor devices, showing a quite different way to traditional thermal annealing. It can effectively remove lattice defects caused by ion implantation [25,26,27]. Additionally, laser annealing is a type of localized annealing that has been widely studied in device preparation because of its advantages, such as fast annealing speed, low heat accumulation, selectable annealing region, localized thermal effect, and good spatial resolution [28,29]. However, this treatment is almost exclusively used in inorganic materials, and laser annealing treatment is hardly used in devices prepared based on organic materials due to their low heat resistance and poor stability [30,31,32,33].

It is known that most organic semiconductor materials are prepared on silicon wafers with a thermally oxidized layer and that when the liquid film is thick enough, the film can be stabilized with gravity. If the thickness of the liquid film is too small, with the help of capillarity, the molecular force overwhelms gravity, and the liquid film is wetted [34,35,36,37]. Dewetting of the material largely destroys the compactness and continuity of the organic semiconductor film arrangement and, therefore, limits the mobility of the device. The difficulties in processing the high-mobility organic semiconductor materials hinder the development of the field and limit their wider applications for various device applications [38,39]. Our recent research revealed that carbon materials (such as graphene and mesoporous carbons) [22,40,41] and organic semiconductors could be processed by lasers.

Here, we designed a new laser annealing method for OFET devices based on the BTBT derivative material Ph-BTBT-12, which solved the problem of the mobility of the devices no longer increasing at a certain temperature due to the dewetting of the film. After surface modification and thermal annealing at 100 °C, the OFET devices showed good stability in the atmosphere, with a maximum mobility of 2.08 cm^2^ V^−1^ s^−1^, while the hole mobility of the devices could be further increased to 2.80 cm^2^ V^−1^ s^−1^ after laser annealing. In addition, our combination of UVO and OTS processing resulted in a significant improvement in device performance compared to devices that received only OTS processing [16,18,42].

## 2. Materials and Methods

### 2.1. Materials

Ph-BTBT-12 (2-Dodecyl-7-phenyl[1]benzothieno[3,2-*b*][1]benzothiophene) and octadecyl trichlorosilane (OTS, >99.5%) were supplied by TCI, Co., Ltd. (Tokyo, Japan) *n*-Heptane (AR, 98%), trichloromethane (AR, ≥99%), and anhydrous ethanol were used to clean OTS residues. The silicon wafers used were from Zhejiang Lijing Co., Ltd. (Hangzhou, China) and the wafer type was p-type heavily doped silicon with 300 nm SiO_2_ as an insulating layer.

### 2.2. Preparation of Thin Films

The film of Ph-BTBT-12 was deposited on the OTS substrate after UV–ozone (UVO) treatment by vacuum deposition thermal evaporation. The Ph-BTBT-12 material was then evaporated using a vacuum pressure of 5 × 10^−4^ Pa. The active layer thickness was 50 nm.

### 2.3. Device Fabrication

The bottom gate top contact OFET devices were prepared on a p-type highly doped silicon wafer with a 300 nm thick SiO_2_ as the gate dielectrics (capacitance per unit area, 10 nF/cm^2^). A 50 nm thick organic semiconductor thin film of the Ph-BTBT-12 was deposited on the SiO_2_/p++ Si substrate treated by UVO and an OTS self-assembled monolayer (SAM). Gold electrodes (50 nm) were deposited by thermal evaporation on the Ph-BTBT-12 film through variable channel shadow masks to define the source and drain contacts. The channel width (W) of the source-drain electrode is 1000 μm, and the channel lengths (L) are 50 μm, respectively. After depositing the gold electrodes, the OFET devices were annealed at 100 °C, 140 °C, and 180 °C (on the same sample). Then, the laser annealing processes were carried out with a 633 nm laser with the power of 5 mW, 20 mW, and 100 mW on a scanning XY stage. The OFET device performances were measured using a Keithley 4200 semiconductor analyzer (Tektronix, Beaverton, OR, USA) at room temperature in atmospheric conditions. The field effect mobility (*μ*), switching ratio (*I*_on_/*I*_off_), and threshold voltage *V*_T_ were extracted by transfer characteristics.

### 2.4. Characterizations

The optical absorption spectrum of the Ph-BTBT-12 thin films was tested with a UV-Vis spectrophotometer (UV-2600, Shimadzu, Kyoto, Japan). The Raman spectra were collected with a Raman spectrometer (LabRAM Aramis, Horiba Jobin Yvon, Palaiseau, France) excited by a 633 nm laser. The morphologies of the Ph-BTBT-12 thin films were obtained using atomic force microscopy (Dimension-3100, Veecco, Welshpool, WA, USA) in the tapping mode. To study the phases of the liquid crystalline Ph-BTBT-12 organic semiconductors, the differential scanning calorimetry (DSC) curves were recorded by a TA Instruments Q100 in a nitrogen atmosphere with a heating rate of 5 °C·min^−1^. The channel sizes tested in this experiment are all W = 1000 μm; L = 50 μm.

## 3. Results

**Figure 1:**
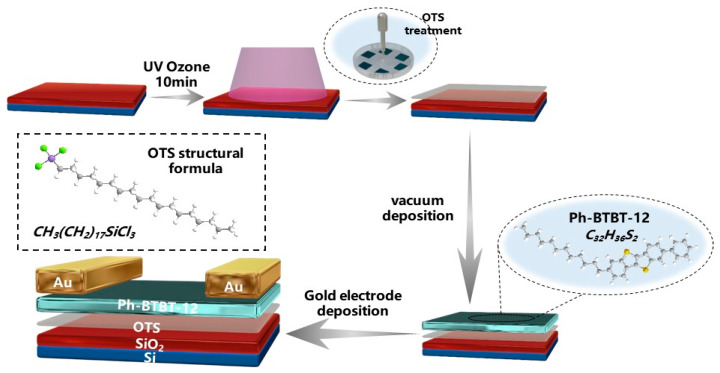
The schematic diagram of OFET device preparation based on Ph-BTBT-12. The substrates were cleaned by acetone and deionized water, respectively, and then passed through ultraviolet–ozone treatment (UVO) for 10 min, followed by OTS surface treatment, (Green indicates Cl, purple indicates Si, gray indicates C, white indicates H, and yellow indicates S).

**Figure 2:**
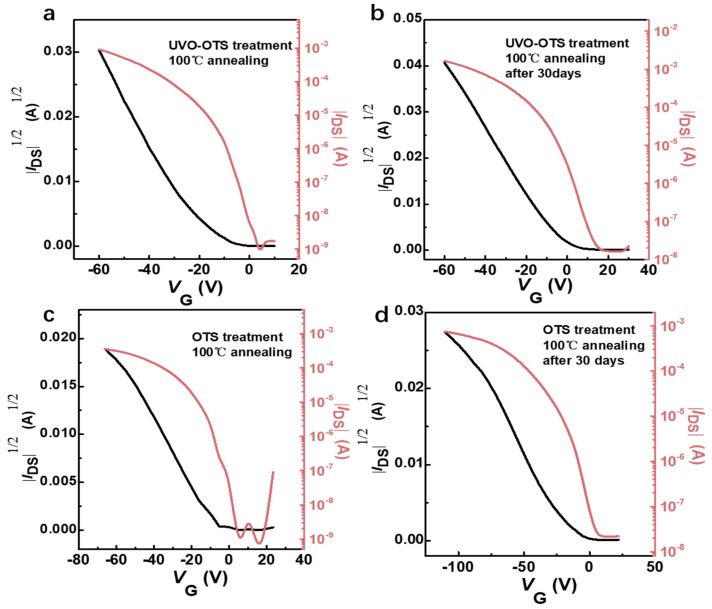
(**a**) Transfer curves of Ph-BTBT-12 devices after ultraviolet–ozone treatment (UVO). (**b**) Transfer curves of Ph-BTBT-12 devices after ultraviolet–ozone treatment (UVO) and re-tested after being placed in a nitrogen glove box environment for 30 days. (**c**) Transfer curves of Ph-BTBT-12 devices after OTS. (**d**) transfer curves of Ph-BTBT-12 devices after OTS treatment and re-tested after 30 days in a nitrogen glove box environment. All the devices were subjected to hot-bench annealing post-treatment with an annealing temperature of 100 °C and time of 10 min prior to testing.

The device preparation process is presented in Figure 1. The surface modification and annealing procedure have significant impacts on the device’s performance. In our experiments, we observed that the devices treated with UVO-OTS maintained a higher hole mobility, similar to the initial test, even after 30 days of being placed in a glove box environment, as compared to the devices treated with other methods, as illustrated in Figure 2. Figure 2a depicts the device prepared after the substrate was treated with UVO-OTS, achieving a calculated hole mobility of 4.2 cm^2^ V^−1^ s^−1^. On the other hand, Figure 2b shows the device transfer curve obtained after 30 days in a nitrogen glove box, with a calculated hole mobility of 4.7 cm^2^ V^−1^ s^−1^. In contrast, Figure 2c,d display the transfer curves of the OTS-treated substrate immediately after preparation and after 30 days of placement, showing a significant decrease in hole mobility to 1.1 cm^2^ V^−1^ s^−1^ and 0.9 cm^2^ V^−1^ s^−1^, respectively. Hence, it is evident that the device’s stability is enhanced after treatment with UVO-OTS. All devices tested and calculated herein were treated with hot bench annealing at 100 °C for 10 min.

**Figure 3:**
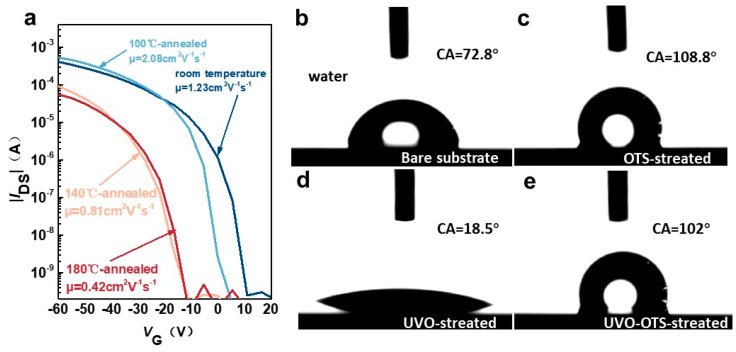
(**a**) The transmission characteristic curves of the device at each annealing temperature. The contact angle of the substrate surface after different treatments: (**b**) exposed silicon wafer; (**c**) OTS treatment; (**d**) UVO treatment; (**e**) UVO-OTS treatment.

The transfer curves corresponding to various annealing conditions were examined, as shown in Figure 3a. Furthermore, the surface contact angle tests derived from various treatments were performed, as shown in Figure 3b–e. With different annealing conditions, the hole mobility of the device is different (Table 1). Self-assembled monolayer (SAM) is commonly applied in surface modification to modify the affinity energy, and the treatment helps in charge transport in organic semiconductors [43,44,45]. Octyltrichlorosilane (OTS) is one typical SAM material and is generally used in the preparation of both organic and inorganic semiconductor devices. The primary effect of OTS as a surface treatment material is to render the device surface hydrophobic. By treating the substrate with OTS, the surface affinity energy can be matched, leading to improved device performance. This is also evident in the results of our experiments [46,47]. Distilled water was dripped on the surface, and its contact angle was then collected to study the substrate surface energy, as shown in Figure 3b–e, which is extremely important for the performance of OFET devices built on this type of substrate. It was discovered that the contact angle of the UVO-OTS-treated substrate surface is lower than that of the OTS-treated substrate and higher than that of the UVO-treated substrate. Also, we discovered that it is not the case that the larger the contact angle, i.e., the more hydrophobic the surface of the device, the better its performance, which will be explained in detail later. With different annealing conditions, the hole mobility of the device is different (Table 1). We carried out the calculation of the average hole mobility of different substrate treatments on the existing data, with a total of 10 groups of samples, and finally obtained the following conclusions: the average hole mobility of the devices treated with OTS is 2.07 cm^2^ V^−1^ s^−1^; the average hole mobility of the devices treated with UVO-OTS is 2.31 cm^2^ V^−1^ s^−1^. Generally, the hole mobility first increases and then decreases with annealing temperature.

### 3.1. Interpretation of Ph-BTBT-12 Material Properties Combined with the Liquid Crystal Phase and Annealing Properties

**Figure 4:**
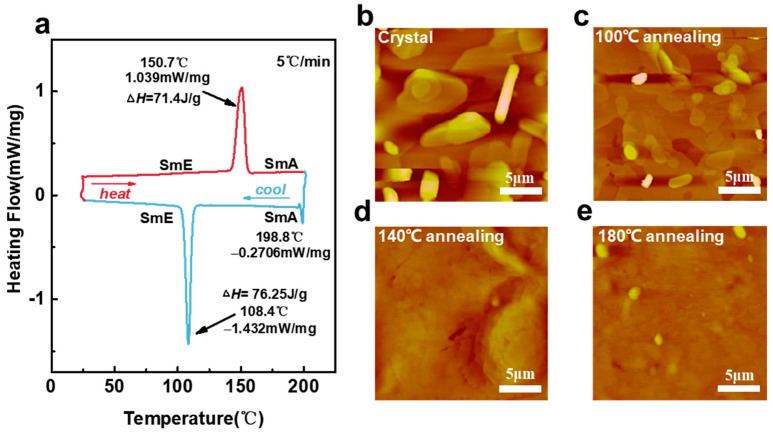
(**a**) The DSC curve of the device (heating rate is 5 °C/min). The AFM images at each annealing temperature (**b**–**e**) and different annealing temperatures correspond to mobilities of 1.23, 2.08, 0.81, and 0.42 cm^2^ V^−1^ s^−1^.

**Figure 5:**
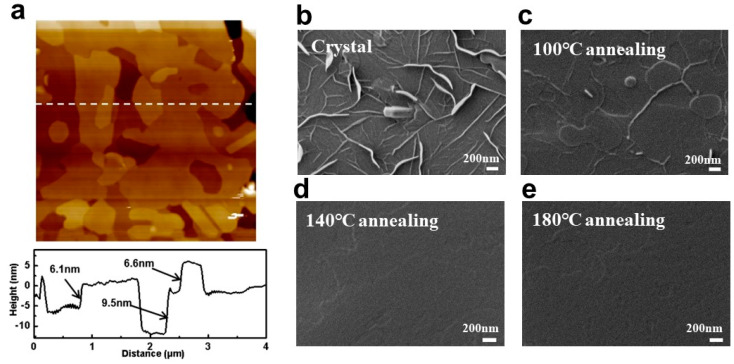
(**a**) AFM image and height diagram of Ph-BTBT-12 material after thermal annealing at 100 °C for 10 min in nitrogen environment of hot station (The dotted line corresponds to the height of the step in the following figure.); (**b**–**e**) SEM images of Ph-BTBT-12 material at four temperatures (room temperature, 100 °C, 140 °C, 180 °C).

We examined the micro-morphology of the surfaces at different annealing temperatures for the same film (100 °C, 140 °C, and 180 °C) by using atomic force microscopy (AFM), as shown in Figure 4b–e. The Ph-BTBT-12 molecules underwent a notable transition, causing the grains to melt and resulting in an overall smooth surface; details on the roughness are obtained in Figure S5a–d. We observed that the rms roughness of the surface decreased by nearly five times immediately after annealing at a temperature of 100 °C. After subjecting the material to annealing at a lower temperature of 140 °C instead of 180 °C, we observed a significant alteration in the step height. The step height actually increased from 11.02 nm to 23.83 nm. Based on this, it is shown that Ph-BTBT-12 can be arranged into a bilayer structure at this temperature. This bilayer structure aligns the two BTBT nuclei in the SmE phase head to head, forming an extended π-conjugated system. The grain size of Ph-BTBT-12 has a close relationship with the device’s performance. As the crystal grain size increases, the crystal domains and the grain boundary become smaller, which leads to huge defects decreasing. (This point is also shown in Figure 5b–e in the following section.) In addition to this, annealing itself has a positive effect on reducing material defects [48]. Hence, the hole transport of the material and the hole mobility of the device can be significantly increased [49,50,51,52]. In addition to this, we compared the mobility of BTBT and related derivatives and compared the results with our results, as shown in Table 2. We found that the Ph-BTBT-12-based devices have high mobility up to 4.8 cm^2^ V^−1^ S^−1^.

Ph-BTBT-12 is an atmospherically stabilized liquid crystalline material, which we characterized and analyzed by DSC (differential scanning calorimetry) (Figure 4a). Detailed molecular length calculations are shown in Figure S2. Higher hole mobility can be obtained due to the highly ordered SmE phase produced after thermal annealing at 100 °C, which increases the active layer charge transfer efficiency of subsequent devices prepared by Ph-BTBT-12 [58]. As the annealing temperature increases (140 °C), the liquid crystal state is still in the SmE phase according to the DSC image, but the hole mobility decreases, which is due to the dewetting effect of the surface of the material that occurs due to the increase in the annealing temperature at 140 °C. More defects are generated on the surface of the device even though the film morphology is still in the SmE direction, and the performance of the film is degraded [59,60,61]. After further annealing to 180 °C, the semiconductor active layer of the device undergoes a phase transition, the liquid crystal phase becomes the SmA phase, the degree of molecular arrangement of the order decreases, and the film dewetting effect is enhanced, which ultimately leads to a further degradation of the device performance [53].

### 3.2. Study on the Change in Laser Annealing on the Properties of Devices Prepared Based on Ph-BTBT-12 Material and Its Optoelectronic Properties

Usually, organic molecules do not wet the silicon wafers with an oxide layer sufficiently, so once the temperature reaches a value above their melting temperature, the crystalline layers of these molecules tend to dewet the substrate. At this point, the viscosity of the interface is low, and dewetting of small molecules is a rather rapid process. Dewetting transforms a previously homogeneous film into an array of droplets or pancakes. According to the above discussion, the device hole mobility is highest at 100 °C and then decreases after subsequent hot bench annealing at a higher temperature (140 °C). The reason can be attributed to the dewetting of the thin films, which affects the hole mobility enhancement of the device. In our study, laser annealing at the channel of the device effectively avoids the dewetting of the liquid crystal film, which results in a significant increase in the hole mobility of the device. In addition, according to the SEM plots (Figure 5b–e) of the material at different temperatures, it is inferred that the change in the hole mobility of the material may also be related to the degree of crystallization of the material, especially in Figure 5b,c. After annealing at high temperatures, the original needle-like room temperature deposition state changes to a regular crystalline state, which may affect the migration of carriers in it and the density of states at the interface with the gold electrode.

Furthermore, the AFM observation in Figure 4c shows that the hot-stand annealing in our experiment promotes the step structure change in Ph-BTBT-12 material from a single-layer step of 3.0 nm to a double-layer step of 6.1 nm, as shown in Figure 5. These changes indicate that the single-layer structure with a thickness of 30.3 Å before heat treatment changes to a double-layer structure with a thickness of 61.2 Å. From the AFM and SEM images, we could identify a preferred vertical alignment orientation in the arrangement of the molecules and a distinct island-like growth pattern in the material deposited at room temperature. These changes in surface roughness were critical in the future optimization of our device’s performance [62]. We discovered that shortly after annealing at 100 °C, the surface’s rms roughness dropped by roughly five times. It was able to discern a preferential vertical alignment orientation in the arrangement of the molecules and a characteristic island-like growth pattern in the material deposited at room temperature using AFM and SEM (Figure 5b–e). After subjecting the material to annealing at a lower temperature of 140 °C instead of 180 °C, we observed a significant alteration in the step height. Contrary to the expected decrease, the step height actually increased from 11.02 nm to 23.83 nm. However, the corresponding root mean square value decreased from 15.55 nm to 6.19 nm, as anticipated. However, there is a decrease in hole mobility due to the material’s dehumidifying effect after undergoing high-temperature annealing. The annealed material’s surface becomes flatter, and the roughness is greatly reduced, which is one of the reasons for the better performance of the annealed device. In general, consistent with the previous description, after the annealing of Ph-BTBT-12 at 100 °C, the grain size of the material decreased, and the grain domains and grain boundaries decreased at the same time in addition to the phase transition (see Figure 5b,c comparison). The defects in the active layer of the device are reduced, and the liquid crystal phase transitions from the state after hot evaporation with low order to the highly ordered SmE phase and from the monolayer structure to the bilayer structure, which makes charge transport easier. The design of materials for OFETs can be enhanced by utilizing a bilayer structure composed of mono-alkylated p-conjugated molecules. This structure represents a quasi-extended large p-conjugated system and offers a new strategy for achieving high mobility without sacrificing solubility. As a result, the hole mobility of the device is significantly improved at 100 °C annealing. When annealed at 140 °C, the material does not undergo the desired transformation, but its hole mobility is significantly reduced, as shown in Figure 4a above. This is because we generally prepare devices on silicon wafers with silicon oxide layers, and the hot-evaporation organic semiconductor films we prepare will be stabilized on the substrate due to the gravity caused by the wetting and thickening of the liquid film, but when the temperature reaches a certain level, the original humidified liquid film undergoes dewetting and becomes thinner, and when it becomes thin to a certain extent, the influence of intermolecular forces will be greater than the influence of gravity on the organic semiconductor film so that the dewetting of the thin films forms an unstable state.

**Figure 6:**
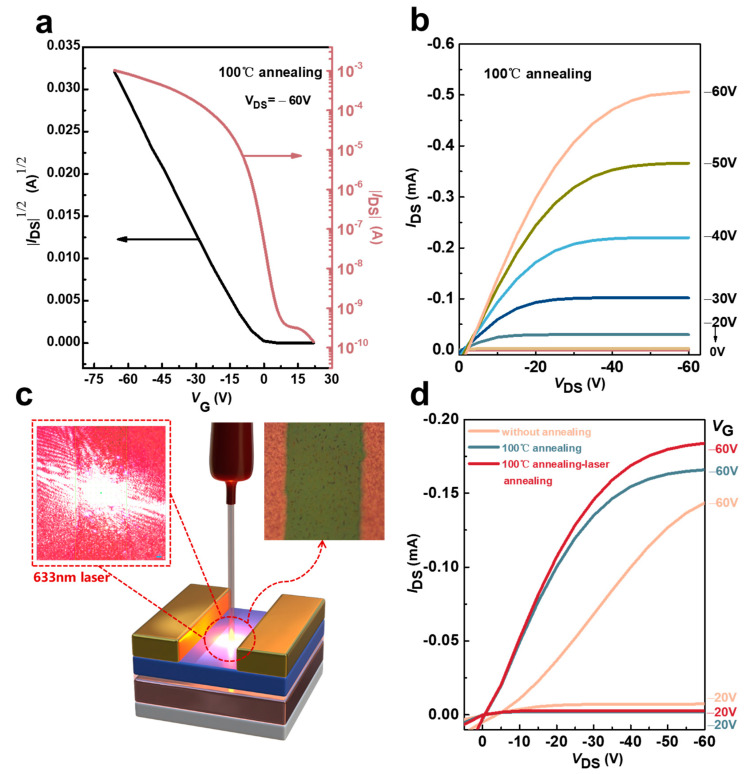
(**a**,**b**) The transition and output curves after annealing with hot table. (**c**) The schematic diagram of the laser annealing process. (**d**) The comparison of output curves among different annealing conditions.

Figure 6 shows the process and performance of the devices with laser treatment. The device was thermally annealed by a heating stage at 100 °C and then cooled down. After that, the selected trench was annealed with a laser with a wavelength of 633 nm. The transfer characteristic curves and output characteristic curves of the selected device before laser annealing and during annealing at 100 °C are shown in Figure 6a,b. The spot diameter is about 40 μm (Figure S7). The output curves of the trench after laser annealing (Figure 6c) were examined and were compared to those of the same trench untreated and annealed only at 100 °C, as shown in Figure 6d. In the subsequent laser treatment, using an optical power meter, we conducted measurements to determine the laser conditions. The 633 nm laser had a laser density of 33.77 mW/cm^2^(this value is the average value obtained after five tests using an optical power meter). In this experiment, during the whole process of using laser annealing, the device did not move on the carrier stage, and only the Raman laser light source moved in the channel. The laser used in Raman mapping is used to scan the channel, and the dot parameters are set to ensure that the processing of the electrode channel of the device is more accurate. A laser spot is punched at every 20 μm interval with a time interval of 1s, and the laser injection time at each point is 1s. The total operating time is determined by the channel length of different components and is generally between 15 and 20 min.

**Figure 7:**
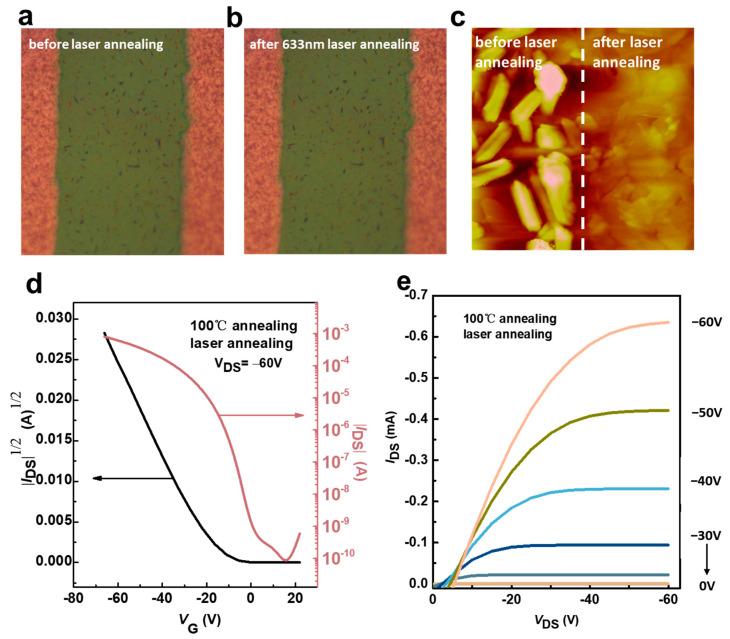
The morphologies at the channel of the device after different laser treatment conditions. (**a**) Without laser treatment, only thermal annealing at 100 °C; (**b**) after laser annealing treatment with a wavelength of 633 nm; (**c**) AFM comparison image before and after laser annealing at room temperature; (**d**,**e**) the output and transfer characteristic curves of laser treatment (633 nm, 100%) after thermal annealing.

In addition, we performed microscopic characterizations of the laser-treated channels, as shown in Figure 7a,b. As shown in Figure 7c, the microscopic AFM images exhibited an obvious height gap between the surfaces of the organic semiconductor film before and after laser annealing at room temperature, and its 3D image is shown in Figure S6. As shown in Figure 7d,e, the hole mobility of the device increases from 2.08 cm^2^ V^−1^ s^−1^ to 2.80 cm^2^ V^−1^ s^−1^ immediately after laser annealing with a wavelength of 633 nm. According to Table 3, the threshold voltage decreases from −21.20 V to −4 V after laser annealing, indicating that the temperature at the channel increases during laser annealing, which reduces the resistance at the active layer channel, which affects the gate voltage, and eventually manifests itself as a decrease in the threshold voltage, and it proves that laser annealing at the channel affects the device performance in our experiments. We hypothesize that laser annealing acting on the surface of organic semiconductors, as shown in Figure 7c, alters the stress action on the surface of the film and increases the intermolecular forces, which enhances the strong electronic coupling between molecules and reduces the intramolecular recombination energy. Laser annealing treatment is capable of generating a specific thermal effect and exhibits selectivity. It can penetrate through the film material directly to the substrate surface beneath the channel material. Due to the localized nature of the laser, it only produces high temperatures and high pressure in specific regions. This thermal effect, in turn, enhances the densification of the thin film material, thereby strengthening the intermolecular force and promoting more orderly and uniform molecular alignment, as depicted in Figure 7c. Furthermore, the thermal effect generated by laser annealing can eliminate defective states on the film’s surface and, to some extent, induce a reaction between the metal electrode and the oxygen in the air, resulting in the formation of oxygen vacancies and an increase in carrier concentration [28,63]. These, in turn, improve the carrier hole mobility of the material and effectively avoid dewetting of the organic semiconductor film at higher annealing temperatures. This means that the device does not need further annealing, which can lead to dewetting of the thin film’s material, and the hole mobility can be improved.

**Figure 8:**
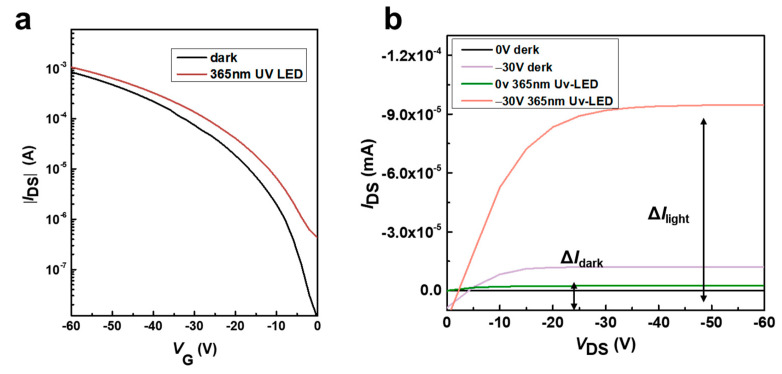
(**a**)Transfer curves of photodetectors in the dark and in the 365 nm LED. (**b**)Typical output curves at zero gate voltage in the dark and under illumination.

Furthermore, the photoelectric response of the device was tested, as shown in Figure 8. It is found that the FET with Ph-BTBT-12 as the active layer has an obvious induction to a UV light source with a wavelength of 365 nm, as shown in Figure 8a [64].

R = ΔI/(P_ill_S) = (I_ph_ − I_dark_)/(P_ill_S) (1)

where P_ill_ is the light intensity, and S is the channel area of the photodetector test device. It is calculated that when V_gs_ = −60 V and the optical power is 15 mW/cm^2^, the maximum value of responsivity R is 8.2 AW^−1^.

The special detectivity D* is an important factor for photodetector devices, and the formula to calculate D* is

D^∗^ = RS^1/2^/(2eI_dark_)^1/2^ (2)

After calculation, the device can achieve a photoelectric detquation rate of 5.0 × 10^11^ cmHz^1/2^ W^−1^. The transmission curve of the photodetector shown in Figure 8b under the illumination of the LED light source with a wavelength of 365 nm also shows that the material has strong response characteristics to the ultraviolet light source.

## 4. Conclusions

In summary, we demonstrate the thermal and laser annealing of a thermotropic liquid crystal organic semiconductor material, Ph-BTBT-12. Ph-BTBT-12 exhibits a highly ordered SmE liquid crystal phase, and its OFET device can reach an optimal hole mobility of 4.8 cm^2^ V^−1^ s^−1^ after annealing at 100 °C. Laser annealing is also applied for the first time to OFET devices prepared based on organic semiconductor films, and the hole mobility can be improved from 2.08 cm^2^ V^−1^ s^−1^ to 2.80 cm^2^ V^−1^ s^−1^ after low-energy laser annealing. Laser annealing effectively avoids the problem of device hole mobility limitations caused by the dewetting of organic semiconductor liquid films. In addition, the phototransistor based on Ph-BTBT-12 has a certain response effect to the ultraviolet wavelength light source (the wavelength is a 365 nm light source), and the optical response characteristic R of the device can reach up to 8.2 AW^−1^, and the responsivity can reach 5.0 × 10^11^ cmHz^1/2^ W^−1^. We also use the UVO-OTS process for surface modification to improve device performance and stability. We surmise that laser annealing can also be applied to organic semiconductor thin-film devices and is expected to solve the unreasonable effect of dewetting organic semiconductor thin films on the devices. In this paper, it was shown that laser annealing can increase the mobility of OFET devices by ~15%, and it can reduce the threshold voltage of the device, thus reducing the device’s turn-on voltage and energy consumption. And due to the characteristics of laser annealing, it can be operated locally, there can be high precision annealing demand device to operate, while laser annealing can reduce the heating and damage of the substrate, and laser irradiation has a direct phototropic effect, leading to rapid crystallization of the film. In addition to inorganic materials such as transparent conductive oxides (TCO), low-power laser annealing technology can also be applied to organic semiconductors and thus has great potential for application in the field of organic electronics.

## Figures and Tables

**Table 1:** Properties of devices prepared with different surface treatments.

	T/°C	μ_sat_ [cm^2^ V^−1^ s^−1^]	I_on_/I_off_	V_th_ [V]
Bare	100	1.08 *	2.34 × 10^5^	−23.13
OTS	100	2.07 **	3.28 × 10^6^	−19.31
UVO-OTS	100	2.31 ***	4.43 × 10^6^	−18.81

* denotes the average value after ten sets of data statistics; the standard deviation is 0.022. ** denotes the average value after ten sets of data statistics; the standard deviation is 0.015. *** denotes the average value after ten sets of data statistics; the standard deviation is 0.019.

**Table 2:** Mobility of devices prepared on the basis of BTBT and its derivatives.

Materials	Preparation Method	μ_sat_ [cm^2^ V^−1^ s^−1^]
Ph-BTBT-10 [53]	spin coating	2.27
TBTBT1 [54]	thermal evaporation	0.24
bis(hydroxy-hexyl)-BTBT [55]	thermal evaporation	0.17
D2-Und-BTBT-Hex [56]	spin coating	0.07
BTBT single-crystals [51]	physical vapor transport deposition	0.032
C8-BTBT [57]	thermal evaporation	2.25
Ph-BTBT-12	thermal evaporation	4.80

**Table 3:** Comparison of device hole mobility before and after laser annealing.

	μ_sat_ [cm^2^ V^−1^ s^−1^]	I_on_/I_off_	V_th_ [V]
Room temperature	0.13	2.34 × 10^5^	−27.3
100 °C annealing	2.08	3.28 × 10^6^	−21.2
Laser annealing	2.80	4.43 × 10^6^	−4.0

## Data Availability

Data are contained within the article and Supplementary Materials.

## Supplementary Materials

The following supporting information can be downloaded at https://www.mdpi.com/article/10.3390/ma17061395/s1, Figure S1: Molecular structure of Ph-BTBT-12 material and its related information; Figure S2: Molecular length of Ph-BTBT-12 material calculated by chem 3D; Figure S3: Transmittance of Ph-BTBT-12 vaporized on a quartz wafer using thermal vapor deposition and annealed at different temperatures; Figure S4: UV-Vis spectra of Ph-BTBT-12 vaporized on a quartz wafer using thermal vaporization after annealing at different temperatures; Figure S5: AFM images of Ph-BTBT-12 films at different annealing temperatures: (a) room temperature; (b) 100 °C; (c) 140 °C; (d) 180 °C; Figure S6: 3D AFM images before and after annealing of the material organ at room temperature; Figure S7: Images of the laser spot in the laser annealing experiment; Figure S8: Histogram of the hole mobility of devices prepared based on Ph-BTBT-12 as an active layer annealed after 100 °C.
