# Supplementary material for: High-Performance Organic Field-Effect Transistors of Liquid Crystalline Organic Semiconductor by Laser Mapping Annealing
Source: Materials (Basel). 2024 Mar 19;17(6):1395. doi: 10.3390/ma17061395 (PMC10972306; doi:10.3390/ma17061395)
Supplement: Supplementary file 1 [file materials-17-01395-s001.zip › materials-2888702-supplementary.pdf]

Supplementary Material

High Performance Organic Field-effect Transistors of Liquid Crystalline Organic Semiconductor by Laser Mapping Annealing

Luying Huang[a, b], Fenghua Liu*[b, c], Jiachen Bao[b, c], Xiaoman Li[b, c], Yunjiao Gu[b, c], and Weiping Wu**[b, c]

[a.] Key Laboratory for Micro-Nano Physics and Technology of Hunan Province, College of Materials Science and Engineering, Hunan University, Changsha, 410082, China.

[b.] Laboratory of Thin Film Optics, Shanghai Institute of Optics and Fine Mechanics, Chinese Academy of Sciences, Shanghai, 201800, China.

[c.] State Key Laboratory of High Field Laser Physics, Chinese Academy of Sciences, Shanghai, 201800, China.

E-mail: fenghualiu@siom.ac.cn, wuwp@siom.ac.cn

Table of Contents

1. **Figure S1**. Molecular structure of Ph-BTBT-12 material and its related information.
2. **Figure S2**. Molecular length of Ph-BTBT-12 material calculated by chem 3D.
3. **Figure S3**. Transmittance of Ph-BTBT-12 vaporized on a quartz wafer using thermal vapor deposition and annealed at different temperatures.
4. **Figure S4**. UV-Vis spectra of Ph-BTBT-12 vaporized on a quartz wafer using thermal vaporization after annealing at different temperatures.
5. **Figure S5**. AFM images of Ph-BTBT-12 films at different annealing temperatures a) room temperature, b) 100°C, c) 140°C, d) 180°C.
6. **Figure S6**. 3D AFM images before and after annealing of the material organ at room temperature.
7. **Figure S7**. Images of the laser spot in the laser annealing experiment.
8. **Figure S8**. Histogram of the mobility of devices prepared based on Ph-BTBT-12 as an active layer annealed after 100 °C.

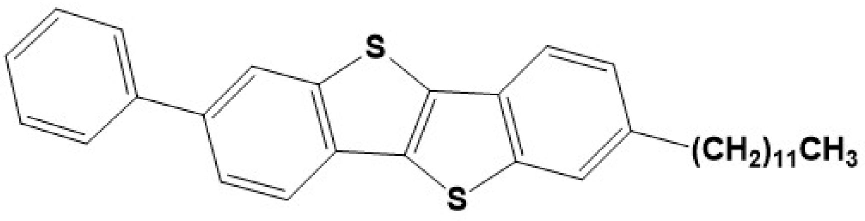

Ph-BTBT-12

3-dodecyl-8-phenylbenzo[*b*]benzo[4,5]thieno[2,3-*d*]thiophene

Chemical Formula: C32H36S2

Exact Mass: 484.23

Molecular Weight: 484.76

m/z: 484.23 (100.0%), 485.23 (36.6%), 486.22 (9.0%), 486.23 (6.4%), 487.22 (3.2%)

Elemental Analysis: C, 79.29; H, 7.49; S, 13.23

Figure S1. Molecular structure of Ph-BTBT-12 material and its related information.

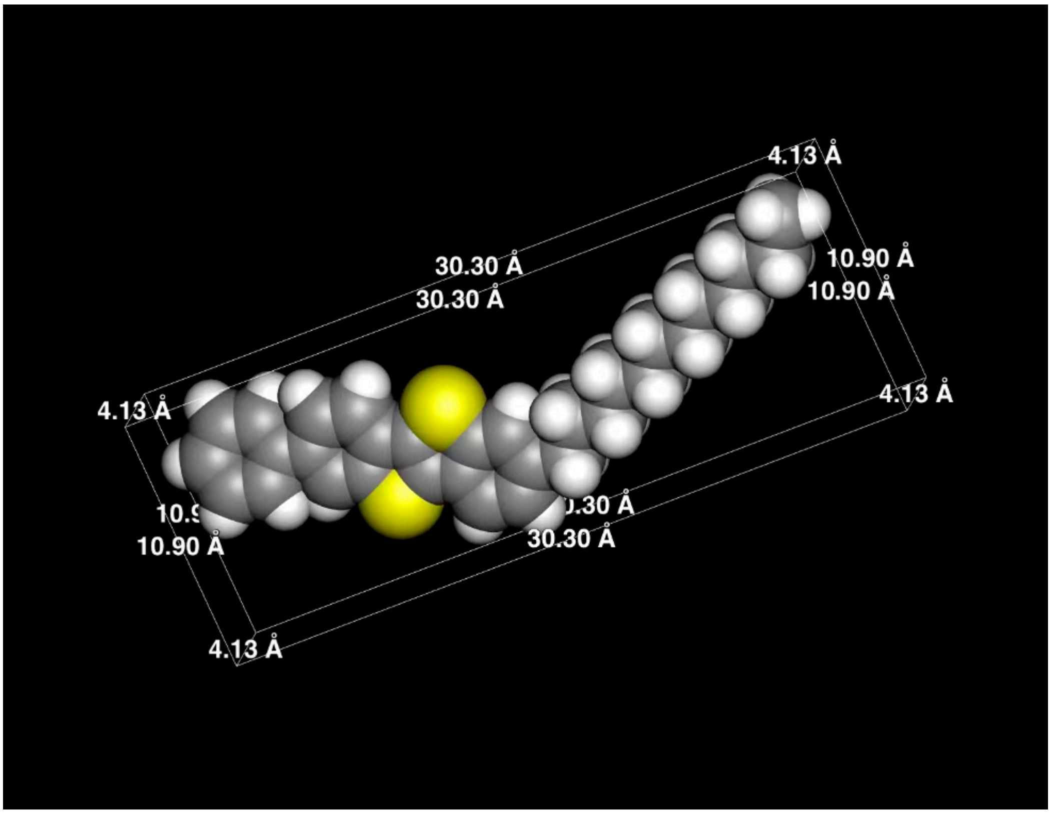

Figure S2. Molecular length of Ph-BTBT-12 material calculated by Chem 3D.

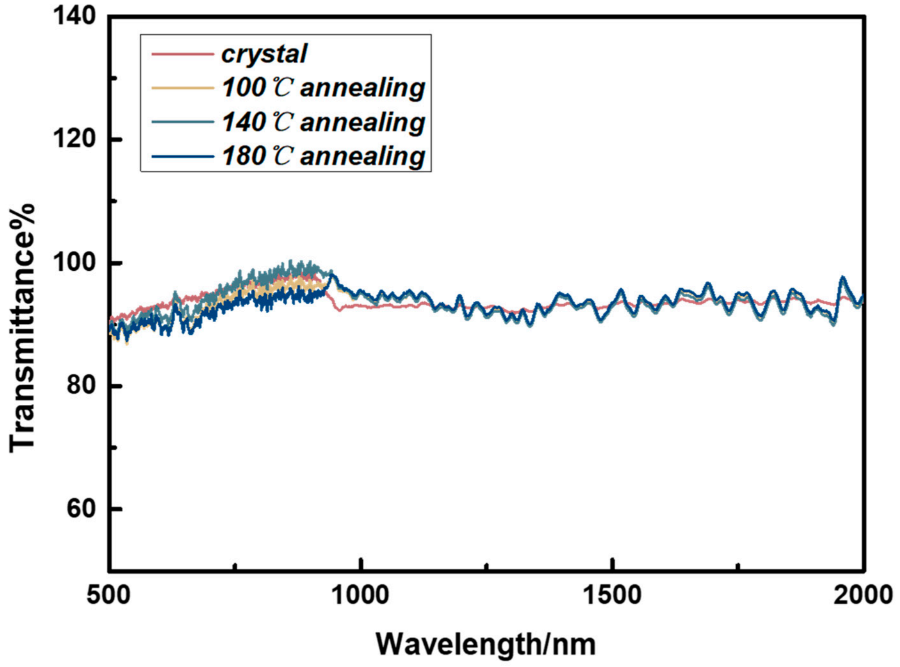

Figure S3. Transmittance of Ph-BTBT-12 vaporized on a quartz wafer using thermal vapor deposition and annealed at different temperatures.

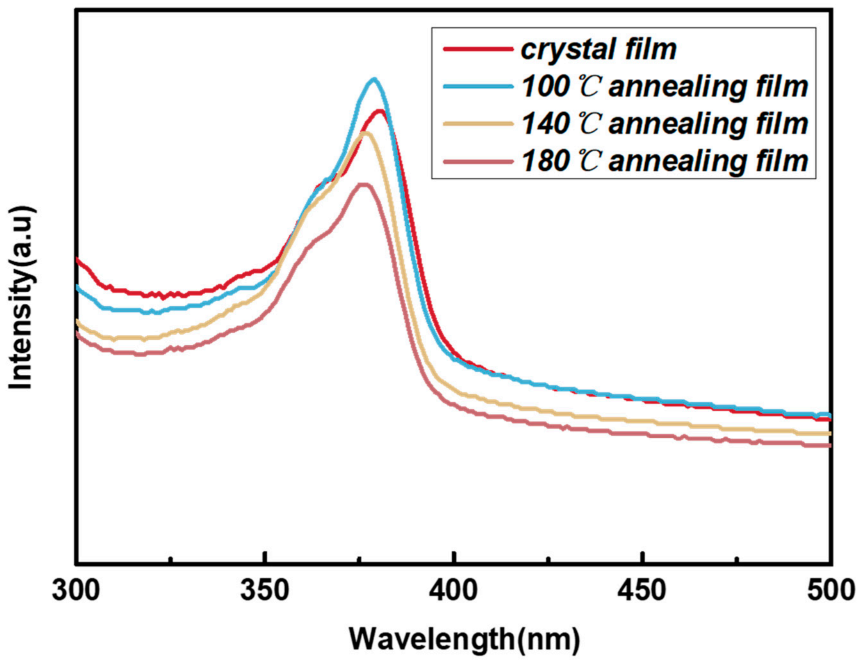

Figure S4. UV-Visible spectra of Ph-BTBT-12 vaporized on a quartz wafer using thermal vaporization after annealing at different temperatures.

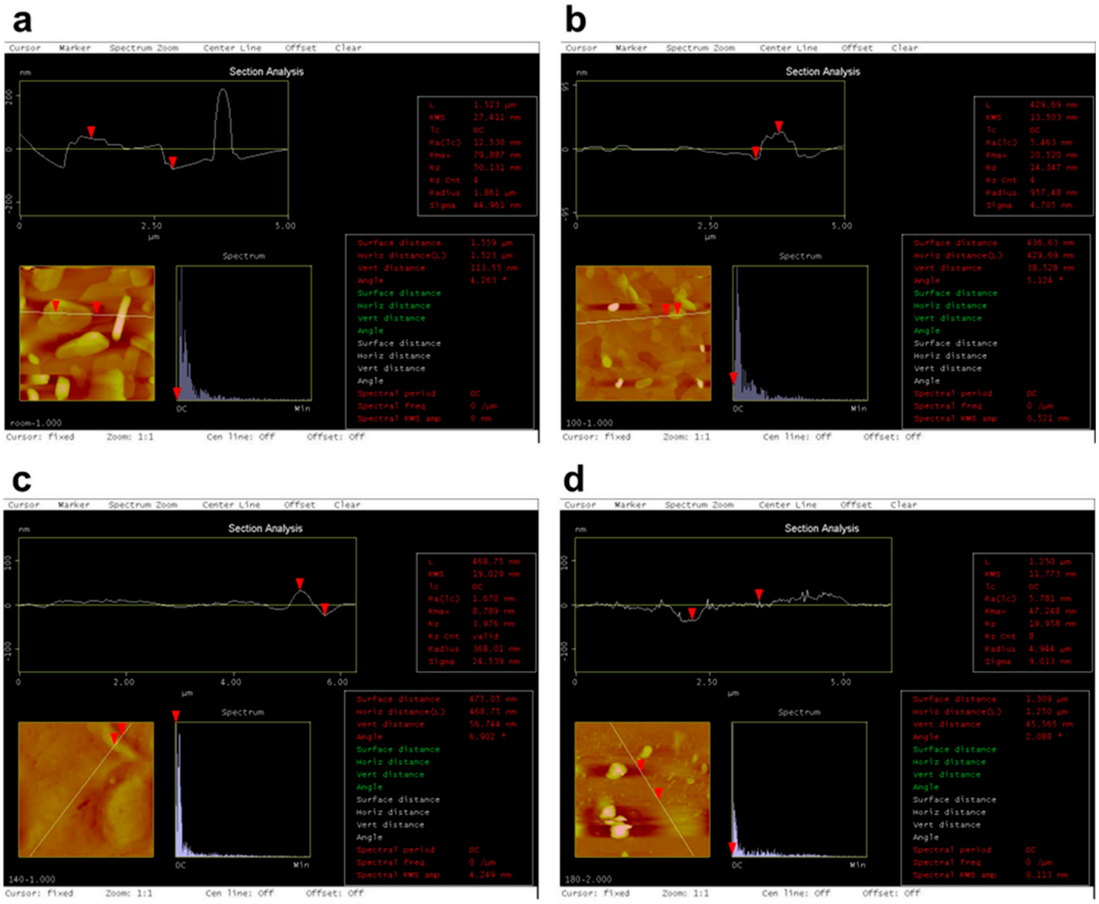

Figure S5. Surface AFM images of Ph-BTBT-12 films at different annealing temperatures a) room temperature, b) 100 °C, c) 140 °C, d) 180 °C.

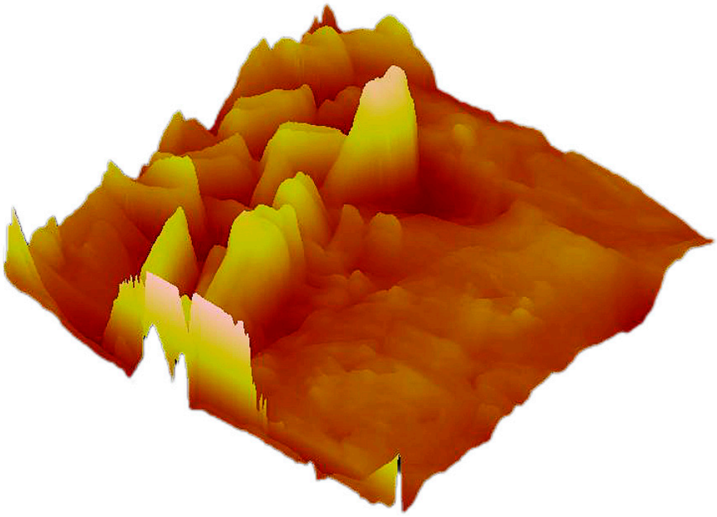

Figure S6. 3D AFM images before and after annealing of the material organ at room temperature.

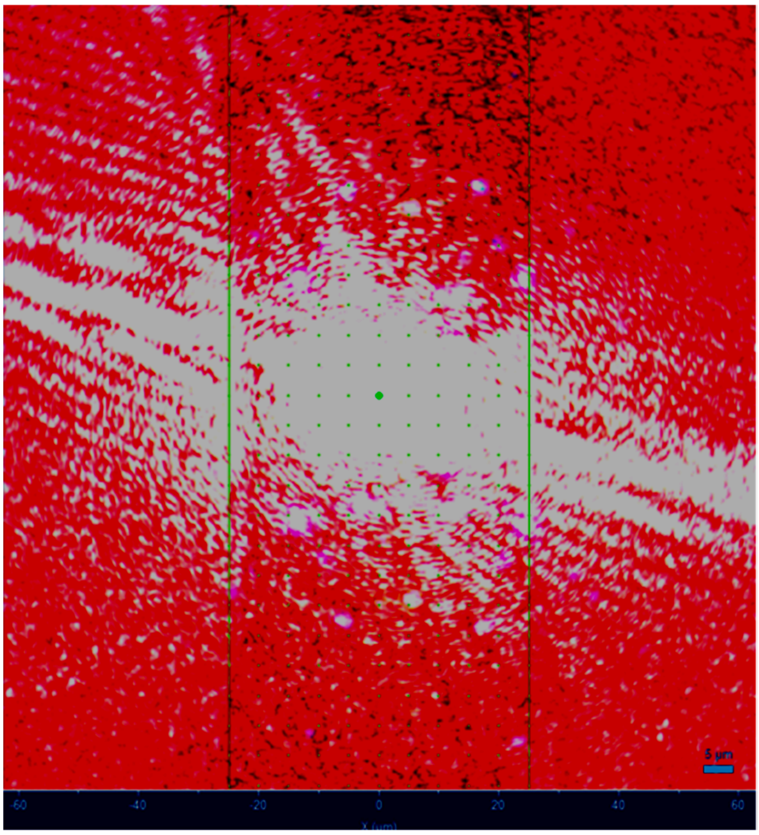

Figure S7. Images of the experiment with the laser turned on, and the approximate diameter range of the laser.

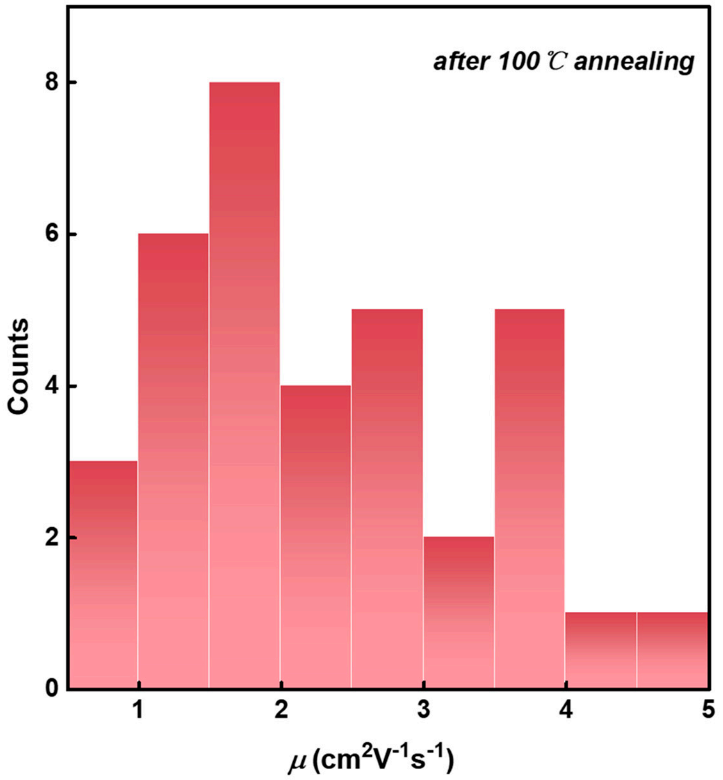

Figure S8. Histogram of the mobility of devices prepared based on Ph-BTBT-12 as an active layer annealed after 100 °C.
